# Potentially inappropriate medication in older participants of the Berlin Aging Study II (BASE-II) – Sex differences and associations with morbidity and medication use

**DOI:** 10.1371/journal.pone.0226511

**Published:** 2019-12-30

**Authors:** Sarah Toepfer, Juliane Bolbrinker, Maximilian König, Elisabeth Steinhagen-Thiessen, Reinhold Kreutz, Ilja Demuth

**Affiliations:** 1 Charité–Universitätsmedizin Berlin, corporate member of Freie Universität Berlin, Humboldt-Universität zu Berlin, and Berlin Institute of Health, Lipid Clinic at the Interdisciplinary Metabolism Center, Berlin, Germany; 2 Charité—Universitätsmedizin Berlin, Corporate Member of Freie Universität Berlin, Humboldt-Universität zu Berlin, and Berlin Institute of Health, Institut für Klinische Pharmakologie und Toxikologie, Berlin, Germany; 3 Medizinische Klinik mit Schwerpunkt Nephrologie und Internistische Intensivmedizin, Charité-Universitätsmedizin, Berlin, Germany; 4 Berlin Insitute of Health Center for Regenerative Therapies”oder ausführlicher „Charité—Universitätsmedizin Berlin, BCRT—Berlin Institute of Health Center for Regenerative Therapies, Berlin, Germany; Istituto Di Ricerche Farmacologiche Mario Negri, ITALY

## Abstract

**Introduction:**

Multimorbidity in advanced age and the need for drug treatment may lead to polypharmacy, while pharmacokinetic and pharmacodynamic changes may increase the risk of adverse drug events (ADEs).

**Objective:**

The aim of this study was to determine the proportion of subjects using potentially inappropriate medication (PIM) in a cohort of older and predominantly healthy adults in relation to polypharmacy and morbidity.

**Methods:**

Cross-sectional data were available from 1,382 study participants (median age 69 years, IQR 67–71, 51.3% females) of the Berlin Aging Study II (BASE-II). PIM was classified according to the EU(7)-PIM and German PRISCUS (representing a subset of the former) list. Polypharmacy was defined as the concomitant use of at least five drugs. A morbidity index (MI) largely based on the Charlson Index was applied to evaluate the morbidity burden.

**Results:**

Overall, 24.1% of the participants were affected by polypharmacy. On average, men used 2 (IQR 1–4) and women 3 drugs (IQR 1–5). According to PRISCUS and EU(7)-PIM, 5.9% and 22.6% of participants received at least one PIM, while use was significantly more prevalent in females (25.5%) compared to males (19.6%) considering EU(7)-PIM (p = 0.01). In addition, morbidity in males receiving PIM according to EU(7)-PIM was higher (median MI 1, IQR 1–3) compared to males without PIM use (median MI 1, IQR 0–2, p<0.001).

**Conclusion:**

PIM use occurred more frequently in women than in men, while it was associated with higher morbidity in males. As expected, EU(7)-PIM identifies more subjects as PIM users than the PRISCUS list but further studies are needed to investigate the differential impact of both lists on ADEs and outcome.

**Key points:**

We found PIM use to be associated with a higher number of regular medications and with increased morbidity. Additionally, we detected a higher prevalence of PIM use in females compared to males, suggesting that women and people needing intensive drug treatment are patient groups, who are particularly affected by PIM use.

## Introduction

Pharmacotherapy in older adults is challenging due to physiological changes and increasing morbidity. Multimorbidity and the need for drug treatment may lead to polypharmacy thereby increasing the risk of adverse drug events (ADEs) [1]. At the same time, pharmacodynamic and pharmacokinetic changes in the elderly, such as decreasing kidney function, additionally raise the risk of ADEs. Thus, the risk of drug-induced toxicity may increase when the dose of renally eliminated drugs is not adjusted to the kidney function. Indeed, about 21% of the BASE-II participants showed a glomerular filtration rate (GFR) <60 mL/min/1.73m^2^, with only a minority of them being aware of their condition [2]. Additionally, polypharmacy has been shown to be associated with impaired kidney function even after controlling for a large range of potential confounders in BASE-II participants [2]. Not only renal function decreases frequently with age, also body composition changes with an increasing proportion of fat tissue and less muscle mass, posing the risk of accumulation of lipophilic drugs such as Diazepam [3]. The use of a drug is defined to be inappropriate, if its potential risk overweighs the clinical benefit [4]. The necessity of drug treatment, as well as availability of safer alternatives are relevant to the classification. Potentially inappropriate medication (PIM) use is associated with ADEs including falls and hospitalization [5]. Many countries developed lists of inappropriate drugs adapted to the country-specific drug market and prescribing behavior.

The German PRISCUS list was created by expert consensus based on a literature review followed by a modified Delphi process [6]. Overall, 83 drugs out of 18 drug classes were rated to be potentially inappropriate for the elderly. For nine drugs upper daily dose limits were specified. The EU(7)-PIM list represents a combined list that was generated based on the German PRISCUS list [6], the French [7], the American [4, 8] and the Canadian list [9] for potentially inappropriate medication. Drugs were classified as PIM, questionable PIM and non-PIM. Overall, 282 drugs from 34 drug classes were defined as inappropriate for older people. Thereof, some medications were restricted to a dose or to the duration of use. The EU(7)-PIM list was created to analyze and compare prescribing habits across European countries, specifically to be used as a screening tool, and for clinical practice [10].

We chose to apply and examine the German PRISCUS list and the EU(7)-PIM list in the current study, because both lists are suitable screening tools for PIM use without requiring clinical information. Since the study was conducted on a German cohort, BASE-II, we wanted to analyze PIM use considering a national PIM list in comparison to the more recently established European screening tool. The conciseness and high specificity [11] of the PRISCUS list to detect inappropriate drug treatment can be seen as a benefit in clinical practice. Unfortunately, PRISCUS has not been updated since 2010. On the other hand, the more recent and comprehensive EU(7)-PIM list might be less specific in predicting adverse outcomes and ADEs [12]. With the current study we aimed to contribute to the assessment of putative additional value of EU(7)-PIM in comparison to the well-established national PRISCUS list.

Both lists set an age threshold of at least 65 years to characterize old people and both lists are explicit [13], meaning they focus on drugs to be avoided in older adults independent of disease or condition. Both lists make suggestions for therapeutic alternatives. All substances of the PRISCUS list are represented in the EU(7)-PIM list, and upper dose limits are equal in both lists.

Amongst others, health insurances databases have been examined to identify the prevalence of inadequate medication according to the PRISCUS list [14–16], but fewer publications used the EU(7)-PIM list to examine PIM use in population based cohorts of older adults [16]. Some studies are limited to prescription data [14, 17]. We considered prescribed drugs as well as over-the-counter medications to assess PIM and polypharmacy. The aim of the current study was to determine the proportion of subjects using PIM in a cohort of older, community-dwelling adults with above-average health. In addition, sex differences and associations with polypharmacy and morbidity were examined.

## Methods

The data for the current analyses were drawn from the Berlin Aging Study II (BASE-II, medical part). Briefly, 2,171 participants, a group of community-dwelling elderly people (60–84 years old, about 75% of the study population) and a control group of younger participants (20–36 years old) were comprehensively investigated. The study protocol contained a structured interview performed by physicians, including a comprehensive history of medication use. For the current study we considered the 1,384 BASE-II participants, which have been at least 65 years old at the time of baseline assessments, which took place between 2009 and 2014. The BASE-II cohort has been described in detail before [18, 19]. All participants gave written informed consent and the Ethics Committee of the Charité-Universitätsmedizin Berlin approved the BASE-II study (approval number EA2/029/09).

Drugs were classified as PIM according to the PRISCUS list [6] and the EU(7)-PIM list [10].

To assess the number of used medications, the drugs participants reported to use regularly were summed up. Beside prescribed drugs, over-the-counter and traditional medications were considered. On-demand medications were not taken into account.

Polypharmacy was defined as the concomitant, regular use of at least five drugs. This definition has already been used to assess polypharmacy in the BASE-II cohort before [2, 20]. Excessive polypharmacy was defined as the concomitant, regular use of at least ten drugs [21].

We evaluated polypharmacy in the context of PIM use to validate the medication history, because being female and using more medications are described to be the most important factors associated with PIM use [5].

A morbidity index (MI) largely based on the categories of the Charlson index was used to evaluate morbidity [22]. As part of the medical examination of BASE-II participants at the Charité—Universitätsmedizin Berlin, diagnoses were obtained through participant reports, with select diagnosis (e.g., diabetes mellitus) being verified by additional blood-laboratory [18]. Diagnoses were used to compute a morbidity index according to the categories of the Charlson index, which is a weighted sum of moderate to severe, mostly chronic physical illnesses, including cardiovascular (e.g., congestive heart failure), cancer (e.g., lymphoma), and metabolic diseases (e.g., diabetes mellitus) [23]. Due to missing data on morbidities, the MI was available for 616 (from the total of 673) males and for 642 (from the total of 709) females.

Calculations were performed with IBM SPSS Statistics, version 25. The Mann-Whitney U test and the Chi-square test were used to compare characteristics between groups. Statistical significance was established *a priori* at p<0.05.

## Results

### Prevalence of PIM use, sex-specific differences

For the current analysis of BASE-II participants aged 65 years and older we excluded two subjects because of missing data on drug treatment, resulting in a total of 1,382 study participants for which cross-sectional data on drug treatment were available. The cohort consisted of 48.7% males (N = 673, median age 69, IQR 67–72) and 51.3% females (N = 709, median age 69, IQR 67–71). According to the PRISCUS list 5.9% (N = 81) of the participants received at least one PIM, with three participants using two PIMs. Regarding the PRISCUS list 4.8% (N = 32) of male participants and 6.9% (N = 49) of female participants used PIM. Concerning the EU(7)-PIM list 19.6% (N = 132) of males and 25.5% (N = 181) of females used PIM. PIM use according to the EU(7)-PIM list was significantly more prevalent in females (p = 0.01).

Altogether, 27 of the total 83 PRISCUS-listed PIMs were found, representing 10 of the 18 drug classes in the list. When considering the EU(7)-PIM list, 22.6% (N = 313) of the 1,382 subjects used at least one PIM. 74 of these 313 participants used two or more PIMs (in total 402 identified substances). 73 out of the 282 listed drugs were detected, representing 23 of the 34 drug classes.

### Most frequently used inappropriate drugs and drug classes

More than half of the PIMs identified belong to the anatomic groups *muscular-skeletal system* and *nervous system* (Table 1). The most frequently used inappropriate substances were diclofenac (N = 59), estrogens (N = 37), ginkgo biloba (N = 23), verapamil (N = 18), acetylsalicylic acid > 325 mg/d (N = 18), glimepiride (N = 16), ibuprofen > 3 x 400 mg/d or for a period longer than one week (N = 16), etoricoxib (N = 10) and venlafaxine (N = 10). From these drugs, etoricoxib is defined as PIM in both lists, whereas the other PIMs were classified as such by the EU(7)-PIM list only. Supplementary Table 1 shows all detected drugs defined as PIM and their anatomic and therapeutic groups.

**Table 1:** Anatomic groups and number of detected drugs defined as PIM.

Anatomic group	Number of PIMs according to PRISCUS (N = 84)	Number of PIMs according to EU(7)-PIM (N = 402)
Alimentary tract and metabolism	-	47
Blood and blood forming organs	-	6
Cardiovascular system	9	64
Genito-urinary system and sex hormones	8	56
Musculo-skeletal system	20	99
Nervous system	38	117
Respiratory system	9	13

Table 1. Number of PIMs of each anatomic group

### Age-specific differences in PIM use

We assigned the participants to four age groups: 65 to 69 years (N = 780), 70 to 74 years (N = 533), 74 to 79 years (N = 58) and 80 to 84 years (N = 11). Because the vast majority of the participants (95%, N = 1,313) was 65 to 74 years old, the age groups 75 to 79 years and 80 to 84 years contained only a small number of participants. Thus, further statistical analyses were only performed for the age groups 65 to 69 years and 70 to 74 years. With respect to the PRISCUS list 5.5% (N = 43) of the participants aged 65 to 69 years and 6.4% (N = 34) of the participants aged 70 to 74 years used at least one PIM. According to the EU(7)-PIM list 21.5% (N = 168) of the participants aged 65 to 69 years and 24.4% (N = 130) of the participants aged 70 to 74 years used at least one PIM. Differences in PIM use with respect to the specified age groups were not statistically significant (p = 0.512 (PRISCUS), p = 0.226 (EU(7)-PIM)).

### Prevalence of polypharmacy in BASE-II, sex-specific differences

Overall, 24.1% (N = 333) of the subjects were affected by polypharmacy and thereof, 2.2% (N = 31) were affected by excessive polypharmacy. The majority (55.3%, N = 765) used one up to four different drugs. 20.5% (N = 284) of the participants stated not to use any regular medication. Table 2 shows the proportion of subjects using 0, 1–4, 5–9, and 10 or more medications separately for men and women. On average, men used 2 drugs (IQR 1–4) and women used 3 drugs (IQR 1–5).

**Table 2:** Proportion of subjects using 0, 1–4, 5–9, and 10 or more drugs.

Number of regular used drugs	Men N = 673	Women N = 709	Overall N = 1382
0	157 (23.3%)	127 (17.9%)	284 (20.5%)
1–4	361 (53.6%)	404 (57.0%)	765 (55.3%)
5–9 (polypharmacy)	143 (21.2%)	159 (22.4%)	302 (21.9%)
≥10 (excessive polypharmacy)	12 (1.8%)	19 (2.7%)	31 (2.2%)

Table 2. Regular used medications were summed up including prescribed drugs, over-the-counter and traditional medications. On-demand medications were excluded.

### Associations of PIM use with the number of medications and the morbidity index (MI)

**Fig 1:**
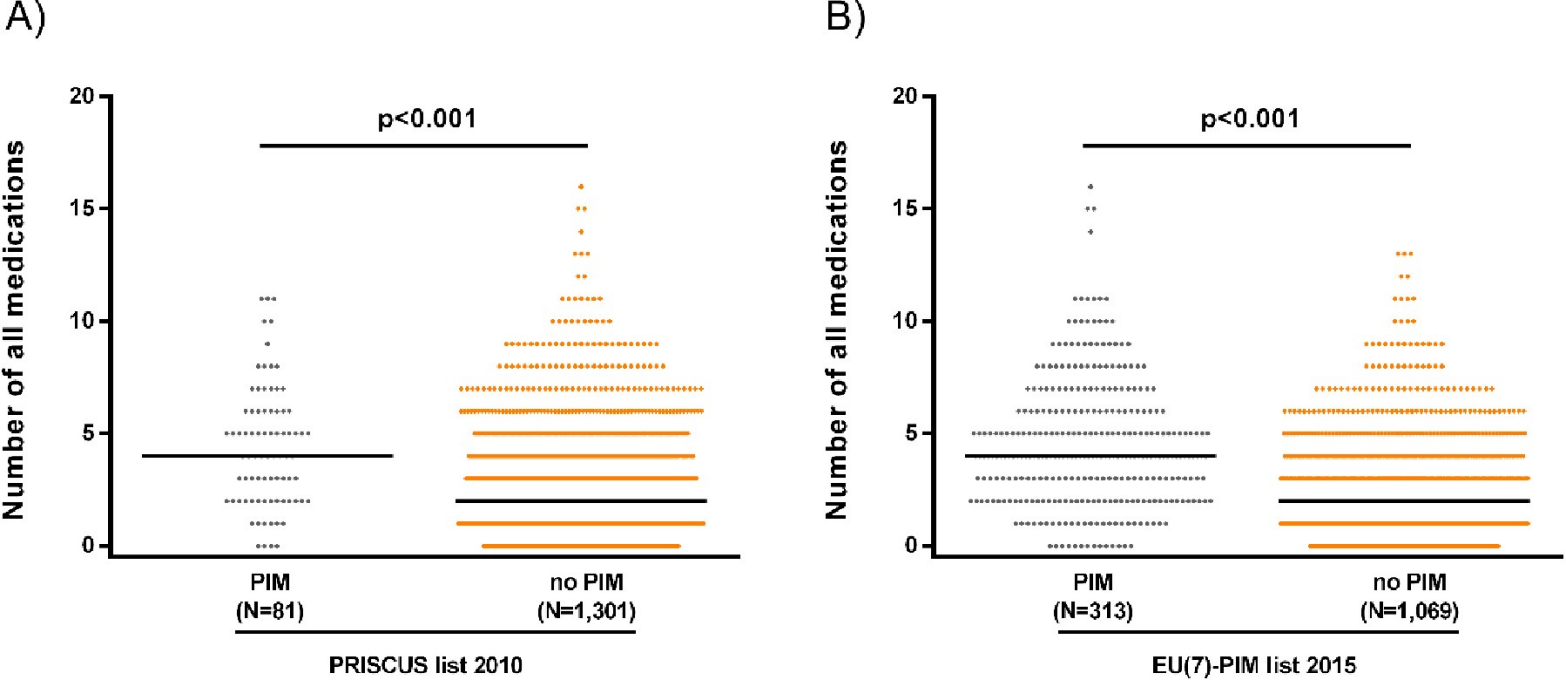
Number of regular medications and PIM use: A) PRISCUS list, B) EU(7)-PIM list. The median number of regular medications is higher in participants using at least one PIM compared to participants without PIM use according to both the PRISCUS criteria and the EU(7)-PIM criteria (Mann-Whitney U test, p<0.001).

The number of regular medications ranged between 0 and 16 different drugs (Fig 1. Number of regular medications and PIM use: A) PRISCUS list, B) EU(7)-PIM list) and was higher in subjects receiving PIM (median 4, IQR 2–6) compared to subjects not using PIM (median 2, IQR 1–4 (PRISCUS), median 2, IQR 0–4 (EU(7)-PIM), p<0.001).

The overall range of the morbidity index (MI) was 0 to 10. The morbidity index of males (N = 616) ranged from 0 to 7, median 1 (IQR 0–2), whereas the morbidity index of females (N = 642) ranged from 0 to 10, median 1 (IQR 0–2). The morbidity index of male participants receiving PIM according to the EU(7)-PIM list was significantly higher (median 1, IQR 1–3) compared to males without PIM use (median 1, IQR 0–2, p<0.001). When excluding the 81 participants using PIM according to both lists and considering only the 232 participants, which are additionally detected by the EU(7)-PIM list, morbidity was still positively associated with PIM use in males (p<0.001). We also selected 81 out of the 313 participants randomly to compensate for the effect of the varying case numbers, and found that there was still a positive association of PIM use with morbidity (p = 0.012). In contrast, an association of PIM use according to the PRISCUS criteria with morbidity could not be shown (p = 0.283).

The morbidity index of females did not differ significantly depending on PIM use.

## Discussion

### Prevalence of PIM use in BASE-II

The identified prevalence of PIM use was 5.9% regarding the PRISCUS list and 22.6% according to the EU(7)-PIM list. As expected, the EU(7)-PIM list identified more study participants as PIM user. The conciseness of the PRISCUS list explains the lower prevalence of PIM use when employing this list in comparison to the detected higher prevalence according to the EU(7)-PIM criteria.

A systematic literature review considering publications using different criteria to define PIM was published in 2015 and reported a prevalence of 22.6% (CI 19.5–26.7%) for potentially inappropriate prescribing in community-dwelling older people across Europe [24]. More than half of the publications considered in this review used the *Beers criteria* [4, 8] to define PIM. This list shows substantial similarities to the EU(7)-PIM list, although the EU(7)-PIM list is more extensive. Studies, which have examined PIM use in a setting similar to ours—community-dwelling people living in Germany—reported a prevalence of PIM use from 12.3% [16] to 22.0% [14] regarding the PRISCUS list and 36.5% to 37.4% [16] considering the EU(7)-PIM list. We found a lower prevalence when applying both the PRISCUS criteria and the EU(7)-PIM criteria. Previous studies have shown, that PIM use has been declining constantly in Germany over time [16, 25], which may indicate that awareness of inadequate medication is rising and affecting prescribing habits. The attitude of patients to medication in general also influences the probability of PIM use. A critical view on medication as well as the ambition to use as few drugs as possible are described to be typical attitudes of non-PIM users [26]. They are more likely to take part in medical examinations and provide relevant information to the medical practitioner [26]. These characteristics apply to most BASE-II participants, which are above average education and actively volunteered to participate in this study [18].

The prevalence of PIM use depends on which criteria were applied. The EU(7)-PIM list detects more PIM than the PRISCUS list, as expected, because it contains 282 drugs, whereas the PRISCUS list contains only 83 substances. A higher sensitivity but a lower specificity of the EU(7)-PIM list to predict adverse drug events in comparison to the *Beers criteria 2015* [27] and the *STOPP criteria 2015* [28, 29] was reported before [12]. In contrast, the PRISCUS list showed a high specificity but a lower sensitivity to detect unfavorable drug treatment [11]. The term PIM already indicates that the listed drugs are not completely contraindicated, but rather *potentially* inappropriate. That means using PIM in an individual case might be indispensable and therefore acceptable. We need to be aware of some cases of individually appropriate medication that are referred to as potentially inappropriate medication in PIM lists, especially when applying more comprehensive lists like the EU(7)-PIM list.

### Most frequently used inappropriate drugs and drug classes

Diclofenac is the most frequently used inappropriate substance (N = 59) we detected. 316.8 million defined daily doses (DDD) were prescribed in Germany in 2014 according to the statutory health insurance report [30]. This analgesic is available in low dosage even without prescription. Diclofenac as well as more selective COX-2 inhibitors such as etoricoxib (10 users) and celecoxib (4 users) should be avoided in older people because their use is limited by a higher incidence of adverse effects particularly in the elderly including an increase in the risk of cardiovascular events [31, 32]. Furthermore, estrogens (N = 37) are a commonly used inappropriate substance group. Although prescription of hormone-replacement preparations has been declining for years, 269 million DDD were still prescribed in 2014 in Germany [30]. Increased mortality from cancer even years after hormone-replacement therapy has been stopped was reported, while no protective effect concerning cardiovascular health could be proven [33]. Ginkgo biloba extracts (N = 23) are traditionally used as over-the-counter (OTC) supplements to prevent or delay cognitive decline, even though evidence for this effect is insufficient [34]. A randomized, double-blind, placebo-controlled clinical trial in subjects aged 75 years or older found no reduction of the incidence rate of dementia or Alzheimer disease, while a non-significant numerical increase in the rate of hemorrhagic strokes was observed in those treated with ginkgo biloba extracts compared to placebo [35]. Acetylsalicylic acid (N = 18) used as an anti-inflammatory drug and ibuprofen (N = 16) when taken more than three times a day or for a period longer than one week, are characterized to be inappropriate because of the increased risk of gastrointestinal bleeding in older adults [36]. Verapamil (N = 18) as a calcium channel blocker should be avoided in older adults, because the inhibition of hepatic cytochrome P450 CYP3A4 and P-glycoprotein influences the metabolism, distribution and bioavailability of other drugs and may lead to interactions, while the drug itself can cause cardiac side effects and obstipation [37]. Glimepiride (N = 16), as well as glibenclamide (N = 4) are commonly used sulfonylureas to treat Type 2 diabetes mellitus and may cause protracted hypoglycemia, especially if the dosage is not adjusted to a reduced kidney function. Sulfonylureas are associated with higher rates of serious hypoglycemia in comparison to metformin, the preferred first‐line medication. Additionally, sulfonylureas induce undesirable weight gain through their insulinotropic effect [38, 39]. Venlafaxine (N = 10) has been associated with an increased risk for various adverse outcomes, among them all-cause mortality, stroke/transient ischaemic attack, upper gastrointestinal bleeding and fractures when compared to other selective serotonin reuptake inhibitors like citalopram or sertraline [40].

### Age- and sex-specific differences in PIM use

The proportion of females with PIM use is significantly higher than the proportion of males using PIM when applying the EU(7)-PIM list (p = 0.01). Female sex is known to be a risk factor for PIM use [5, 16]. Reasons are the use of estrogens, which affects only women, but also the more frequently use of sleep-inducing drugs, antidepressants and analgesics due to a higher prevalence of anxiety disorders, depression and sleep disorders in women than in men [41].

We did not detect a significant difference regarding the proportion of PIM users in the age group 65 to 69 years compared to the age group 70 to 74 years. In previous studies, age was inconsistently associated with PIM use. An increase of PIM use with age has been described [16], as well as a lower prevalence of PIM use in older age groups (75 to 84 years) in comparison to younger age groups (65 to 74 years) [42, 43].

### Associations with polypharmacy and morbidity

Almost a quarter of the participants aged 65 years and older were affected by polypharmacy. Thereof, 2.2% were affected by excessive polypharmacy. A prevalence of 21.1% for polypharmacy in BASE-II in an analysis including people younger than 65 years at baseline assessment was reported before [20]. Studies involving community-dwelling adults have shown even higher prevalences of 36.1 to 39.1% (polypharmacy) and 7.7 to 8.9% (excessive polypharmacy) [44, 45]. A large Dutch study described an increase in polypharmacy over time from 1999 to 2014 [46]. Another Danish study described increasing polypharmacy from 2000 to 2011 but a stable prevalence from 2011 to 2014 [45]. Living longer typically results in the manifestation of chronic disease. Increased life expectancy is a reason for a higher prevalence of polypharmacy. The development of clinical guidelines, which provide evidence-based recommendation for drug use, especially regarding prevention and treatment of cardiovascular and metabolic diseases, additionally increases the number of prescribed drugs per patient [47].

Similar to our finding of a comparably low prevalence of PIM use in BASE-II, also the prevalence of polypharmacy was lower than in other similar cohorts. A reason for this may be that study participants were healthier than the general population of Germany [18] and therefore needed less drug treatment. The median number of drugs is significantly higher in people using at least one PIM. A recent systematic review identified polypharmacy, in addition to female sex, as a determinant for potentially inappropriate medication use in community-dwelling older adults in the United States [5]. We confirmed earlier results with respect to the association of PIM use with female sex and with the number of medications, indicating the validity of the physician assessed medication history captured by an interview.

We found PIM use to be associated with increased morbidity in males regarding the EU(7)-PIM list and obtained the same result when excluding participants affected by PIM use employing the PRISCUS criteria. Associations of PIM use with frailty [48], increased risk of adverse drug events [49] and hospitalization [50] have already been described. Therefore, the association of morbidity and PIM use seems plausible. Although no association of PIM use according to the PRISCUS criteria and morbidity could be shown, we detected PIM use to be associated with morbidity when applying the EU(7)-PIM criteria, even when excluding the PIM users also detected by the PRISCUS list or when selecting 81 PIM users (the number detected by PRISCUS) randomly from EU(7)-PIM users. These results indicate an additional benefit of the EU(7)-PIM list, which should be subject of further investigation.

### Strengths and limitations

In addition to prescription drugs, we considered OTC and traditional medication to capture PIM use. That distinguishes our analyses from previous studies which investigated health insurance data [25], in which only prescription is considered. For example, OTC sleep aids containing diphenhydramine or doxylamine have anticholinergic properties that increase the risk of falling, hangover effects and cognitive impairments in older people [51]. Although a large cohort was investigated here, the results can only be transferred to the German general population with caution because the BASE-II cohort was not a random sample and participants were positively selected as to education, health and cognition [18]. Further studies considering PIM use according to different lists in relation to adverse outcomes are needed, and are planned for the cohort studied here when follow-up data are available.

### Conclusion

In summary, PIM use in BASE-II was more prevalent in women compared to men, was associated with higher morbidity in males and with a higher number of medications in both sexes when the EU(7)-PIM list was considered. As expected, the latter identifies more subjects with PIM use than the shorter German PRISCUS list. Further studies should examine which list is superior in terms of balancing specificity and sensitivity to detect inappropriate medication causing ADEs.

## Supporting information

S1 Table Anatomic groups, therapeutic groups and number of detected drugs defined as PIM. S1 Table shows anatomic groups, therapeutic groups and the number of detected drugs defined as PIM.
(PDF)

S2 Table Proportion of females, median age, median number of regular medications and Morbidity index (MI) of PIM users and non-PIM users. S2 Table shows the proportion of females, median age, median number of regular medications and Morbidity index (MI) of PIM users and non-PIM users.
(PDF)
