# Supplementary material for: Potentially inappropriate medication in older participants of the Berlin Aging Study II (BASE-II) – Sex differences and associations with morbidity and medication use
Source: PLoS One. 2019 Dec 30;14(12):e0226511. doi: 10.1371/journal.pone.0226511 (PMC6936809; doi:10.1371/journal.pone.0226511)
Supplement: S1 Table — shows anatomic groups, therapeutic groups and the number of detected drugs defined as PIM. (PDF) [file pone.0226511.s001.pdf]

Supporting Table 1. Anatomic groups, therapeutic groups and number of detected drugs defined as PIM

<table>
<tr><th>Anatomic group</th><th>Therapeutic group (drug class)</th><th>Drugs defined as PIM by PRISCUS
N=84</th><th>Drugs defined as PIM by EU(7)-PIM
N=402</th></tr>
<tr><td rowspan="4">Alimentary tract and metabolism, N=47</td><td>Drugs for acid-related disorders, N=6</td><td>-</td><td>Ranitidine, N=6</td></tr>
<tr><td>Drugs for functional gastrointestinal disorders, N=8</td><td>-</td><td>Mebeverine, N=2,
Metoclopramide, N=5,
Alizapride, N=1</td></tr>
<tr><td>Laxatives, N=3</td><td>-</td><td>Sodium picosulfate, N=2,
Aloe, N=1</td></tr>
<tr><td>Drugs used in diabetes, N=30</td><td>-</td><td>Glibenclamide, N=4,
Glimepiride, N=16,
Sitagliptine, N=8,
Vildagliptine, N=2</td></tr>
<tr><td>Blood and blood forming organs, N=6</td><td>Antithrombotic agents, N=6</td><td>-</td><td>Dipyridamole, N=1,
Dabigatran, N=3,
Rivaroxaban, N=2</td></tr>
<tr><td rowspan="11">Cardiovascular system,
N=64</td><td rowspan="2">Cardiac therapy, N=13</td><td colspan="2">Digoxin, N=1,
Flecainide, N=2</td></tr>
<tr><td>-</td><td>Amiodarone, N=2,
Dronedarone, N=1,
Digitoxin, N=7</td></tr>
<tr><td rowspan="2">Antihypertensives, N=10</td><td colspan="2">Doxazosin, N=4</td></tr>
<tr><td>-</td><td>Moxonidine, N=6</td></tr>
<tr><td>Diuretics, N=8</td><td>-</td><td>Spironolactone >25 mg/d, N=8</td></tr>
<tr><td>Vasoprotectives, N=1</td><td>-</td><td>Aescin, N=1</td></tr>
<tr><td rowspan="2">Beta-blocking agents, N=5</td><td colspan="2">Sotalol, N=1</td></tr>
<tr><td></td><td>Propranolol, N=4</td></tr>
<tr><td rowspan="2">Calcium channel blockers, N=24</td><td colspan="2">Nifedipine (non-sustained-release), N=1</td></tr>
<tr><td>-</td><td>Nifedipine, N=6,
Verapamil, N=18</td></tr>
<tr><td>Lipid modifiying agents, N=3</td><td>-</td><td>Niacin, N=3</td></tr>
<tr><td rowspan="3">Genito-urinary system and sex hormones,
N=56</td><td>Sex hormones and modulator of the genital system, N=37</td><td>-</td><td>Estrogens (oral), N=37</td></tr>
<tr><td rowspan="2">Urologicals, N=19</td><td colspan="2">Oxybutynine, N=3,
Solifenacin, N=5</td></tr>
<tr><td></td><td>Trospium, N=4,
Darifenacin, N=4,
Fesoterodin, N=1,
Tolterodine, N=2</td></tr>
<tr><td rowspan="2">Muscular-skleletal system, N=99</td><td rowspan="2">Anti-inflammatory and anti-rheumatic products, N=96</td><td colspan="2">Indometacin, N=3,
Meloxicam, N=3,
Ketoprofen, N=1,
Etoricoxib, N=10</td></tr>
<tr><td>-</td><td>Diclofenac, N=59,
Celecoxib, N=4,</td></tr>
</table>

			Ibuprofen >3x400 mg/d or for a period longer than one week, N=16
	Muscle relaxants, N=3	-	Tetrazepam, N=3
Nervous system, N=117	Analgesics, N=29	-	Acetylsalicylic acid >325mg/d, N=18,<br>Triptanes, N=6,<br>Tramadol, N=5
	Antiepileptics, N=3	-	Clonazepam, N=3
	Antiparkinson drugs, N=8	-	Ropinirole, N=1,<br>Pramipexole, N=3,<br>Cabergoline, N=1,<br>Piribedil, N=1,<br>Rotigotine, N=2
	Psycholeptics, N=19	Olanzapine (>10 mg/d), N=1,<br>Zolpidem (>5 mg/d), N=6,<br>Zopiclone (>3.75 mg/d), N=3,<br>Lormetazepam (>0.5 mg/d), N=1,<br>Diazepam, N=4,<br>Bromazepam, N=1	
		-	Lithium, N=3
	Psychoanaleptics, N=58	Clomipramine, N=1,<br>Trimipramine, N=3,<br>Amitriptyline, N=6,<br>Doxepin, N=5,<br>Fluoxetine, N=6,<br>Piracetam, N=1	
		-	Venlafaxine, N=10,<br>Ginkgo biloba, N=23,<br>Bupropion, N=1,<br>Paroxetine, N=2
Respiratory system, N=13	Drugs for obstructive airway diseases, N=3	-	Theophylline, N=3
	Antihistamines for systemic use, N=10	Diphenhydramine, N=4,<br>Doxylamine, N=4,<br>Dimetindene, N=1	
		-	Promethazine, N=1

Supporting Table 1 shows anatomic groups, therapeutic groups and the number of detected drugs defined as PIM
