# Supplementary material for: Potentially inappropriate medication in older participants of the Berlin Aging Study II (BASE-II) – Sex differences and associations with morbidity and medication use
Source: PLoS One. 2019 Dec 30;14(12):e0226511. doi: 10.1371/journal.pone.0226511 (PMC6936809; doi:10.1371/journal.pone.0226511)
Supplement: S2 Table — shows the proportion of females, median age, median number of regular medications and Morbidity index (MI) of PIM users and non-PIM users. (PDF) [file pone.0226511.s002.pdf]

Supporting Table 2. Proportion of females, median age, median number of regular medications and Morbidity index (MI) of PIM users and non-PIM users

Variable	PRISCUS list		EU(7)-PIM list	
	PIM users N=81	Non-PIM users N=1301	PIM users N=313	Non-PIM users N=1069
Female sex	49 (60.5%)	660 (50.7%)	181 (57.8%)	528 (49.4%)
Median age (years)	69 (IQR 68-71.5)	69 (IQR 67-71)	69 (IQR 67-71)	69 (IQR 67-71)
Median number of regular medications	4 (IQR 2-6)	2 (IQR 1-4)	4 (IQR 2-6)	2 (IQR 0-4)
Morbidity index (MI)<br>• Number of subjects included<br>• Median	77<br>1 (IQR 0-2)	1181<br>1 (IQR 0-2)	295<br>1 (IQR 0-2)	963<br>1 (IQR 0-2)

Supporting Table 2 shows the proportion of females, median age, median number of regular medications and Morbidity index (MI) of PIM users and non-PIM users
